# Bilateral Severe Sterile Inflammation with Hypopyon after Simultaneous Intravitreal Triamcinolone Acetonide and Aflibercept Injection in a Patient with Bilateral Marked Rubeosis Associated with Ocular Ischemic Syndrome

**DOI:** 10.1155/2017/5123963

**Published:** 2017-03-13

**Authors:** Ceren Durmaz Engin, Ziya Ayhan, Süleyman Men, Aylin Yaman, A. Osman Saatci

**Affiliations:** ^1^Department of Ophthalmology, Dokuz Eylul University, Izmir, Turkey; ^2^Department of Radiology, Dokuz Eylul University, Izmir, Turkey

## Abstract

We report the clinical course of a diabetic patient with bilateral cataract and rubeosis in association with ocular ischemic syndrome and initially treated him with simultaneous intravitreal 2 mg aflibercept and 2 mg triamcinolone acetonide injection at the same setting prior to planned cataract surgery and further photocoagulation. However, sterile anterior segment inflammation characterized by hypopyon occurred four days apart in OU. Right eye developed the sterile inflammation at the third postinjection day and the left eye developed the sterile inflammation at the seventh postinjection day (two days after the uneventful cataract surgery with intraocular lens implantation) without any pain or significant redness. Vitreous biopsy taken during the right phacovitrectomy was negative for any microbial contamination. Both eyes were treated successfully with intensive topical prednisolone acetate with a relatively good visual outcome. It is likely that underlying ocular ischemic syndrome might have facilitated the formation of sterile inflammation as blood-aqueous barrier disruption and flare have already been present.

## 1. Introduction

Ocular ischemic syndrome (OIS) is characterized with hypoperfusion related anterior and posterior segment changes in association with the occlusion or stenosis of carotid artery [1, 2]. Mild iritis, flare, and posterior synechia are the main anterior segment findings whereas flare is more marked than the cellular inflammatory reaction. Retinal vascular changes such as narrowed retinal arteries, dilated retinal veins, scattered retinal hemorrhages, and cotton wool spots are among the posterior segment findings.

We report the clinical outcome of a patient with bilateral rubeosis due to ocular ischemic syndrome who developed bilateral sterile severe inflammation with hypopyon following the bilateral simultaneous intravitreal injection of aflibercept and triamcinolone acetonide at the same setting.

## 2. Case Report

A 66-year-old man with a history of type 2 diabetes of 20 years' duration, high blood pressure, and previous larynx cancer surgery was referred to us for the treatment of bilateral severe rubeosis iridis and cataract. Referring physician performed two sessions of laser photocoagulation with the diagnosis of proliferative diabetic retinopathy in the left eye but no laser could be performed in the right eye due to presence of a dense cataract. He was put on a fixed combination of timolol maleate and dorzolamide twice a day in OU.

**Figure 1:**
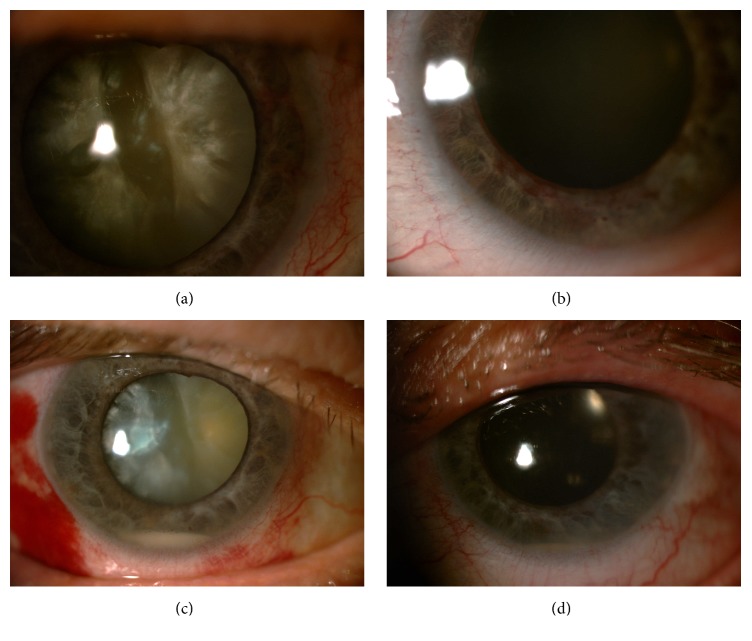
Color anterior segment picture: (a) right eye, whitish cataract and rubeosis iridis at the presentation; (b) left eye, nuclear sclerosis and rubeosis iridis at the presentation; (c) right eye, hypopyon, regressed rubeotic vessels, and the whitish cataract at the third postinjection day; (d) left eye, mild corneal edema, hypopyon, and intraocular lens at the second postcataract surgery (seventh postinjection day).

On our examination, visual acuity was hand motions in OD and counting fingers at 3 meters in OS. Slit-lamp examination revealed severe rubeosis iridis with an almost white cataract in OD and 4+ nuclear sclerosis in OS (Figures 1(a) and 1(b)). Intraocular pressure was 16 mm Hg with the Goldmann applanation tonometer in OU. While the right fundus could not be visualized, there were scattered laser scars 360° and a dry looking macula in OS with the indirect ophthalmoscopy. The right posterior segment was normal with the B-scan ultrasonography. We initially felt that bilateral rubeosis iridis was due to proliferative diabetic retinopathy and planned to complete the laser photocoagulation rapidly after the removal of cataract and intraocular lens implantation in OU. In order to obtain a relatively quiet eye prior to cataract surgery we bilaterally injected intravitreal 2 mg aflibercept (Eylea; Regeneron, Tarrytown, NY, USA) and 2 mg triamcinolone acetonide (Sinakort A, İbrahim Etem Ulagay, Turkey) simultaneously at the same setting in the operating theatre as we had a good experience with the simultaneous anti-VEGF and steroid injection in eyes with severe diabetic macular edema and proliferative diabetic retinopathy [3]. At the third postinjection day, there was one mm hypopyon with 2+ cells in the right anterior chamber (Figure 1(c)) and the left eye was quiet with markedly regressed rubeosis. Most noteworthily, the patient had no pain or any other relevant complaints in the OD. We elected to follow the right eye with hourly prednisolone acetate drops and cyclopentolate 1% three times a day and hospitalized the patient. The next day, the hypopyon was markedly diminished in the right eye and no further change was noted in the left eye. We performed an uneventful cataract surgery and intraocular lens implantation in the OS five days after the intravitreal injection. However, the left eye developed one mm hypopyon with 2+ cells again without any pain at the second postoperative day (Figure 1(d)).

**Figure 2:**
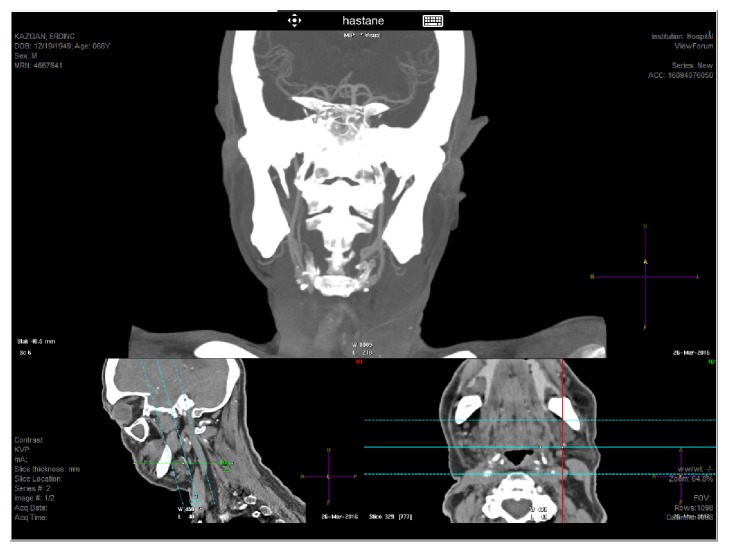
Coronal oblique reformatted computed tomography angiographic image through the neck shows occlusion of both common carotid arteries. Both internal and external carotid arteries are patent, however, filling via collateral flow. Intracranial carotid circulation and posterior circulation are patent.

The left eye was also put on hourly prednisolone acetate drops and 1% cyclopentolate tid. We immediately performed a phacovitrectomy in the OD to obtain a vitreous sample for the microbial investigation, remove the already present dense cataract, and perform the endophotocoagulation at the same time. A vitreous sample was obtained prior to start of 23 g vitrectomy and then two other 23 g sclerotomies were done. Following the completion of uneventful phacoemulsification and intraocular lens implantation, core vitrectomy was performed. The vitreous looked almost totally clear and thereby no antibiotics were injected as we were planning to do so beforehand. The severity of retinopathy was mild to moderate looking and there was no visible neovascularization. Anyhow, a 360° panretinal photocoagulation was performed. Direct microscopic evaluation of the vitreous specimen was normal and no growth was noted in the vitreous culture. Meanwhile, we suspected the presence of OIS and thereby decided to investigate the carotid artery system. Computerized tomography angiographic evaluation showed that both internal and external carotid arteries were perfused only by collaterals and there was marked stenosis in the common carotid arteries (Figure 2).

**Figure 3:**
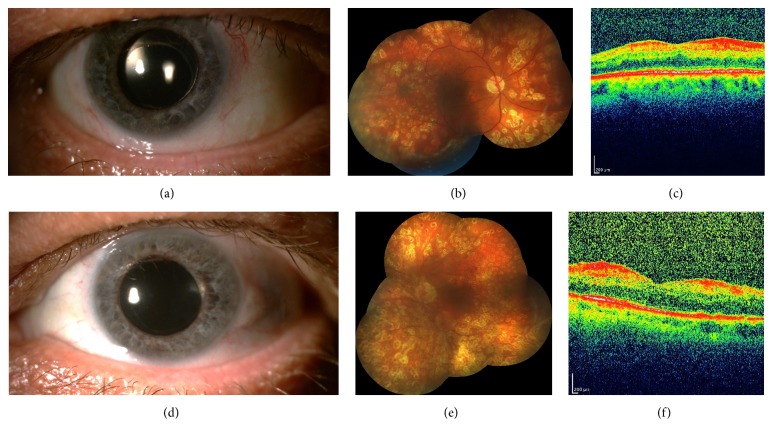
(a) Color anterior segment picture of the right eye with a quiet anterior segment and intraocular lens; (b) right composite fundus picture showing the laser scars scattered 360 degrees and a normal-looking macula; (c) right OCT scan depicting the normal macular contour; (d) color anterior segment picture of the left eye with a quiet anterior segment and intraocular lens; (e) left composite color fundus picture demonstrating the 360 degrees' scattered laser scars and a normal-looking macula; (f) left OCT scan depicting the normal macula.

The patient was put on 100 mg acetyl salicylic acid upon the neurology consultation. We suggested that the event was bilateral sterile inflammation triggered by the intravitreal injection of aflibercept and/or triamcinolone acetonide in the presence of severe rubeosis iridis due to ocular ischemic syndrome. A week after the left cataract surgery and five days after the right phacovitrectomy both anterior segments became quieter with an hourly prednisolone acetate drops and topical steroid was tapered slowly. Two weeks later, additional laser photocoagulation was administered to complete the panretinal photocoagulation in OS. Three months later, the best-corrected visual acuity was 4/10 in OD and 1/10 in OS. Both anterior segments were quiet (Figures 3(a) and 3(b)). Intraocular pressure was normal in OU. Both posterior segments had full panretinal photocoagulation with a normal macula architecture (Figures 3(c), 3(d), 3(e), and 3(f)).

## 3. Discussion

Sterile endophthalmitis may occur almost after intravitreal injection of any drug but when it happens, the clinician faces a great diagnostic and treatment challenge as it is difficult to differentiate the event from the infectious endophthalmitis [4].

Sterile inflammation following the intravitreal aflibercept injection is previously reported but its cause is still unknown [5–10]. The American Society of Retina Specialists' therapeutic surveillance subcommittee first reported 15 cases of aflibercept related sterile inflammation [6] and later expanded this series to 56 cases from the practices throughout the United States between December 2011 and February 2014 [10]. Most cases presented with an initial visual deterioration and intraocular inflammation without prominent redness, severe pain, or hypopyon and median time to presentation was two days after the injection but it could be as late as 30 days. However, there was a case presenting with a hypopyon. Thirty-seven cases (66%) were treated with topical corticosteroids and/or observation alone. The mean time to resolution was 28,6 days. No difference in visual outcome was detected in patients who received only topical corticosteroids and/or observation with the eyes treated with intravitreal antibiotics or vitrectomy. In none of the cases with a vitreous tap a microbial agent could be identified. Most cases had a history of prior aflibercept injection and even some cases were successfully rechallenged with aflibercept suggesting that the sterile inflammation might not be related to patient specific immunologic response. Goldberg et al. [7] reviewed the medical records of 20 patients who presented with noninfectious inflammation after the intravitreal aflibercept injection out of 5356 aflibercept injections. The patients presented one to 13 days after the injections (median, 3 days). While three of 20 (15%) had pain and two of 20 (10%) had conjunctival injection one patient developed also a hypopyon. Patients on average had received six prior aflibercept injections (range 0–16). Four patients were subsequently rechallenged with aflibercept and only one developed inflammation again after five additional aflibercept injections. All patients were managed with frequent topical steroids. The overall incidence of inflammation after intravitreal aflibercept injection was found to be 20 of 5356 injections (0,37%) or 19 of 844 patients (2,25%). Fine et al. [9] published a single-center retrospective study on 28 cases of intraocular inflammation noted after a total of 5905 aflibercept injections among the 1660 patients. Vitreous culture and subsequent antibiotic injection were performed in eight cases and all cultures were negative. The remaining patients received only topical corticosteroids. The authors calculated the frequency of inflammation as 0,47% per injection following the aflibercept injections.

Sterile endophthalmitis after the intravitreal injection of triamcinolone acetonide occurs between 0,20 and 12,3% of the injections [11–15]. Most of the time, it occurs within the first three days of injection and the patients do not complain of eye pain and hypopyon does not develop. Aetiology of the sterile inflammation is not still fully understood [4]. Several suggestions had been made. Contamination of the triamcinolone vials with endotoxins has been postulated [11]. A toxic effect of the triamcinolone itself as well as the preservatives present in the vial such as benzyl alcohol, polysorbate 80, and carboxymethylcellulose sodium has also been incriminated [16, 17]. However removal of benzyl alcohol has not eliminated the occurrence of sterile inflammation [18]. Moreover, commercial form of preservative-free triamcinolone acetonide was also reported to create a sterile inflammation [19]. Some authors suggested that triamcinolone acetonide related sterile inflammation might be induced by the migration of macrophages induced by the crystals [12, 20]. Although sterile inflammation seen after the intravitreal triamcinolone acetonide injection is generally thought to have a benign course some reported cases had a very poor visual and anatomic outcome [21].

In the present case, we concluded that the bilateral severe inflammation together with the hypopyon was sterile as there was no clue for infectious aetiology such as the presence of clear vitreous observed during the right phacovitrectomy and the negative vitreous culture. In addition, both affected eyes responded pretty well to topical frequent corticosteroid administration. Both simultaneously administered aflibercept and/or triamcinolone acetonide might be the factor in the occurrence of sterile inflammation either alone or together. It is likely that underlying ocular ischemic syndrome might facilitate the formation of sterile inflammation as ocular ischemic syndrome can be associated with a blood-aqueous barrier disruption and flare.
